# Cod Liver Oil Supplement Consumption and Health: Cross-sectional Results from the EPIC-Norfolk Cohort Study

**DOI:** 10.3390/nu6104320

**Published:** 2014-10-16

**Authors:** Marleen A.H. Lentjes, Ailsa A. Welch, Angela A. Mulligan, Robert N. Luben, Nicholas J. Wareham, Kay-Tee Khaw

**Affiliations:** 1 Department of Public Health and Primary Care, Strangeways Research Laboratories, University of Cambridge, 2 Worts Causeway, Cambridge CB1 8RN, UK; E-Mails: angela.mulligan@phpc.cam.ac.uk (A.A.M.); robert.luben@phpc.cam.ac.uk (R.N.L.); 2 Department of Population Health and Primary Care, Norwich Medical School, University of East Anglia, Norwich Research Park, Norwich NR4 7TJ, UK; E-Mail: a.welch@uea.ac.uk; 3 Medical Research Council, Epidemiology Unit, Institute of Metabolic Science, Addenbrooke’s Hospital, Cambridge CB2 0QQ, UK; E-Mail: nick.wareham@mrc-epid.cam.ac.uk; 4 Clinical Gerontology Unit, School of Clinical Medicine, University of Cambridge, Addenbrooke’s Hospital, Cambridge CB2 2QQ, UK; E-Mail: kk101@medschl.cam.ac.uk

**Keywords:** dietary supplement, cod liver oil, socio-demographics, health, confounding, cardiovascular disease

## Abstract

Supplement users (SU) make healthy lifestyle choices; on the other hand, SU report more medical conditions. We hypothesised that cod liver oil (CLO) consumers are similar to non-supplement users, since CLO use might originate from historical motives, *i.e.*, rickets prevention, and not health consciousness. CLO consumers were studied in order to identify possible confounders, such as confounding by indication. The European Prospective Investigation into Cancer (EPIC) investigates causes of chronic disease. The participants were 25,639 men and women, aged 40–79 years, recruited from general practices in Norfolk, East-Anglia (UK). Participants completed questionnaires and a health examination between 1993 and 1998. Supplement use was measured using 7-day diet diaries. CLO was the most common supplement used, more prevalent among women and associated with not smoking, higher physical activity level and more favourable eating habits. SU had a higher occurrence of benign growths and bone-related diseases, but CLO was negatively associated with cardiovascular-related conditions. Although the results of SU characteristics in EPIC-Norfolk are comparable with studies worldwide, the CLO group is different from SU in general. Confounding by indication takes place and will need to be taken into account when analysing prospective associations of CLO use with fracture risk and cardiovascular diseases.

## 1. Introduction

Since the 19th century, cod liver (CLO), for its source of vitamin D, has been used as one of the remedies to cure rickets [1]. It has been the most commonly used supplement in the UK for decades [2,3,4,5,6]. In EPIC-Norfolk, 32% of men and 45% of women used dietary supplements between 1993 and 1998 [7] with nearly 25% of all participants consuming CLO [8]. Special interest in CLO supplement use is warranted for several reasons. Firstly, for its nutrients, CLO contains eicosapentaenoic acid and docosahexaenoic acid, which in observational studies have been negatively associated with several cancer sites [9,10]; on the other hand, meta-analyses of trial and/or cohort data have shown no effect of these omega-3 fatty acids in supplement form on cardiovascular disease [11,12]. CLO also provides vitamins A, D and E of which vitamin D prevents osteomalacia and has been associated with osteoporosis [13]; while chronic intake above 1500 µg/day of vitamin A might increase the risk of fractures [14]. Secondly, methodological reasons: there is no such person as “a supplement user” [5,15], supplement users (SU) are heterogeneous and ignoring these differences can lead to bias [16]. These differences are not only due to lifestyle [17], but also to what is referred to as “confounding by indication” [5,18,19]. Meaning that certain co-morbidities make the use of certain dietary supplements more likely, which, if not taken into account, could lead to the conclusion that there is an association between the exposure, *i.e.*, dietary supplements, and outcome (e.g., fracture) when in fact the co-morbidity (e.g., osteoporosis) is merely an indication for, or increases, supplement use.

Before public health messages can be formulated to encourage or discourage CLO use, also in the light of possible harmful effects when overdosed [20], a careful analysis of eating habits and other possible confounders in CLO consumers will have to precede this [16]. Supplement use in general in the United Kingdom [3,4,5,21], Europe [22], the United States [23] and Australia [24,25] has been associated with socio-demographic factors such as being a woman, being older, having a higher socio-economic status; behaviour-wise, SU exercise more, smoke less, eat more healthily and have a lower body mass index (BMI). Whether CLO consumers share the same characteristics as SU in general, and as a result would confound the association found between CLO consumption and health, requires further study. The high proportion of CLO consumers, as well as the detailed information collected in this aging cohort, puts EPIC-Norfolk in a position to study such associations.

This paper describes the socio-demographics, eating habits, anthropometry and self-reported health of NSU and SU in EPIC-Norfolk, with a special focus on the most commonly consumed supplement: CLO.

## 2. Methods

### 2.1. Study Design and Participant Selection

This study was conducted according to the guidelines laid down in the Declaration of Helsinki and all procedures were approved by the Norfolk District Health Authority Ethics Committee. Written informed consent was obtained from all participants. The study started in 1993 with participants aged between 40 and 79 years. Participants lived in the Norfolk area of East Anglia and were recruited from general practitioners’ (GP) age-sex registers [26]. Of the 77,630 invited participants, 30,447 gave their informed consent and received a Health and Lifestyle Questionnaire. Of this group, 25,639 attended a health examination at their GP-clinic and were given a 7-day Diet Diary (7dDD).

### 2.2. Data Collection

The Health and lifestyle Questionnaire was sent to the participants in advance of their GP clinic appointment. Participants were asked about the following: smoking habits (never, former or current smoker); final level of education obtained (no qualifications, O-level, A-level, Degree or equivalent); current profession, from which socio-economic class was derived (unskilled, semi-skilled, skilled manual, skilled non-manual, managerial or professional); marital status (married, single, widowed, divorced or separated); a validated physical activity score combining occupational and recreational physical activity (active, moderately active, moderately inactive, inactive) [27]; and self-reported illnesses, such as cardiovascular diseases, diabetes, cancer and osteoporosis, measured by the question: “Has the doctor ever told you that you have any of the following”. The participant’s postcode was linked to the Townsend residential area deprivation score. This score identifies material deprivation by using four components: unemployment, non-car ownership, non-house ownership and overcrowding, *i.e.*, the number of people who live per room in a house [28].

Participants were taken through the research protocol by a trained nurse [26]. During the health examination, weight (kg) and height (cm) were measured from which Body Mass Index (BMI) was calculated (kg/m^2^).

A 7dDD was handed out at the health examination [29,30]. This diary was a 45-page, A5 booklet, with detailed instructions regarding how food and drink should be recorded, as well as seventeen series of colour photos, depicting portions of food items on plates in increasing quantities. The nurse completed a 24-h diet recall as a means of instruction (*n* = 25,507; 99%). The remainder of the 7dDD was completed at the participant’s home, 23,638 (92%) of the participants completed more than one day.

The 7dDD ended with general questions, referred to as the “Back Of Diary (BOD)”, and was completed by 23,309 (91%) participants [8]. The BOD included the question relating to supplement use (“Please name any vitamins, minerals or other food supplements taken on each day of last week”). If this question was left open, crossed out or answered with “no/none”, then participants were categorised as NSU; however, if participants had recorded any supplements taken, they were categorised as SU. Kappa-statistics with instruments recalling supplement use over the past year in EPIC-Norfolk ranged from 0.72–0.78 [7]. Supplements were coded according to the Vitamin and Minerals Supplement (ViMiS) system described in detail elsewhere [8]. Summarised, supplements were grouped into 45 distinct groups of which CLO was one. This group included CLO or any other type of fish oil, and CLO supplements combined with multivitamins or with, for example, evening primrose oil in the *same* capsule. For the purpose of this analysis, participants who reported medication containing vitamins and/or minerals without further supplement use were classified as NSU.

The 7dDD were entered by trained data-entry clerks using a program called DINER, Data Into Nutrients for Epidemiological Research [30] and checked and calculated by nutritionists using DINERMO [31]. Alcohol intake in grams was divided by 8 to obtain the number of units in alcoholic beverages. Food group data were calculated by summing the weight of each food item consumed, belonging to either fruit, vegetables, red, white or processed meat or white and fatty fish, as well as the percentage contribution to these food groups from composite food items (e.g., Beef stew including vegetables) [31]. These food groups were chosen because of established associations with cancer and cardiovascular risk factors [32].

### 2.3. Statistical Analysis

The characteristics of participants were compared using two different groupings. First, NSU *vs.* SU, followed by two SU subgroups in order to elucidate possible confounding factors for CLO users: SU+CLO, participants who used CLO *or* supplements where cod liver oil/fish oil was an ingredient, also when used in combination with non-CLO supplements (*i.e.*, multiple supplement users of which at least one contained CLO); SU-CLO, participants who consumed one or more supplements that did not contain CLO.

Both comparisons were firstly carried out without adjustment, stratified by sex, using the Chi-squared statistic, followed by multivariable binary (SU *vs.* NSU) and multinomial (SU+CLO *vs.* NSU and SU-CLO *vs.* NSU) logistic regression to compare these groups adjusted for all presented socio-demographic variables.

Differences in food consumption between NSU and SU groups were tested using the Mann-Whitney U and Kruskal-Wallis statistic. Associations between self-reported illnesses and supplement use were adjusted for age using multinomial logistic regression, with supplement use as the dependent variable (NSU/SU+CLO/SU-CLO). Analyses were performed using SPSS v19 (IBM Corp., Armonk, NY, USA). *p*-values below 0.05 were considered significant.

## 3. Results

### 3.1. Supplement Consumption

Out of 23,039 participants who answered the BOD, 3253 (31.7%) of men (*n* = 10,247) and 5736 (44.8%) of women (*n* = 12,792) used a supplement (χ^2^ (1) = 410.01, *p* < 0.001). A total of 5262 and 10,732 supplements were consumed by men and women respectively. For both men and women, CLO was the most commonly consumed supplement (43% and 32% respectively), followed by garlic (12%) and multivitamins (11%) for men and multivitamins (11%) and evening primrose oil (10%) for women. For CLO supplements, 94% of men and 96% of women used these on a daily basis compared to 89% and 90% respectively for non-CLO supplements. CLO supplements were consumed by 22% of men and 26% of women. Only 10% of men consumed supplements that did not contain CLO, compared to 19% of women.

### 3.2. Socio-Demographic Characteristics of Supplement Users

Supplement use in general was associated with sex-dependent characteristics (see columns NSU and SU in Table 1 for men and Table 2 for women). Male SU were older than NSU, whereas female SU completed higher levels of education than female NSU. Marital status was not, and Townsend score only weakly, associated with supplement use. All other characteristics had, in general, stronger associations among women compared to men. In summary, supplement use indicated a healthier lifestyle and higher socio-economic class.

The characteristics of the participants who consumed a supplement that contained CLO (SU+CLO) *vs.* NSU and participants who consumed other types of supplements (SU-CLO) *vs.* NSU (see Table 1 and Table 2), resulted in stronger associations with socio-demographic characteristics with supplement use, especially among women. Notably, younger age in women was strongly associated with the SU-CLO category, similarly for higher education level; however, such associations among SU+CLO were not present. In men, not being married as well as a higher education level, though not associated with supplement use in general, was associated with SU-CLO.

Results of the fully adjusted analysis (NSU *vs.* SU) are to be found in Table 1 and Table 2 (see column SU *vs.* NSU). 3.6% of the participants were lost due to missing values for one or more variables (*n* = 22,205). For supplement use in general, results remained the same as in the unadjusted analysis, except for the area deprivation score in both sexes. Smoking had the strongest association with supplement use, decreasing the odds of supplement use with 41% in men and 29% in women; followed by physical inactivity in men and winter season and lower social class in women. The analysis was repeated with sex in the model (data not shown) and showed a significant independent effect of sex on supplement use in general (OR = 0.54; 95% CI: 0.51–0.58).

Multinomial logistic regression compared NSU with the two SU subgroups (SU+CLO and SU-CLO, see last two columns in Table 1 and Table 2). Results were similar compared to the unadjusted analysis, with exception of education level among men and area deprivation score in both sexes, which lost their significance. Strongest associations were again seen for current smoking; other variables, particularly social class and education, were more strongly associated with the SU-CLO group and not with the SU+CLO group when compared to supplement use in general.

**Table 1:** Characteristics of European Prospective Investigation into Cancer (EPIC)-Norfolk participants (men only) according to supplement status (Non-supplement User (NSU)/Supplement User (SU)) and supplement subgroup (NSU/SU+cod liver oil (CLO)/SU-CLO). Analyses are shown unadjusted (Chi-squared test). Logistic regression (NSU/SU) and multinomial logistic regression (NSU/SU+CLO/SU-CLO) were adjusted for all variables in this table (*n* = 9943). Boldly printed OR were statistically significant findings.

*MEN*	*NSU N*	*%*	*SU N*	*%*	*SU+CLO N*	*%*	*SU-CLO N*	*%*	*SU vs. NSU OR*	*95% CI*	*SU+CLO vs. NSU OR*	*95% CI*	*SU-CLO vs. NSU OR*	*95% CI*
Age	6994		3253		2215		1038							
<=50 years	1696	24.2	528	16.2	285	12.9	243	23.4	**1.15**	1.12–1.18	**1.21**	1.17–1.24	**1.05**	1.01–1.09
>50–60 years	2199	31.4	950	29.2	649	29.3	301	29.0	OR represents the % change in odds of being					
>60–70 years	2120	30.3	1218	37.4	876	39.5	342	32.9	a SU for every 5 year increment in age					
>70 years	979	14.0	557	17.1	405	18.3	152	14.6						
	χ^2^ (3) = 118.51, *p* < 0.001				χ^2^ (6) = 170.43, *p* < 0.001									
Marital status	6967		3227		2198		1029							
Married	6110	87.7	2839	88.0	1956	89.0	883	85.8	Ref		Ref		Ref	
Not married ^a^	857	12.3	388	12.0	242	11.0	146	14.2	1.01	0.88–1.15	0.88	0.75–1.03	**1.30**	1.07–1.58
	χ^2^ (1) = 0.16, n.s.				χ^2^ (2) = 6.76, *p* < 0.05									
Social class	6881		3197		2174		1023							
Non-Manual ^b^	3935	57.2	1961	61.3	1265	58.2	696	68.0	Ref		Ref		Ref	
Manual ^c^	2946	42.8	1236	38.7	909	41.8	327	32.0	**0.87**	0.79–0.95	1.00	0.90–1.11	**0.63**	0.55–0.74
	χ^2^ (1) = 15.50, *p* < 0.001				χ^2^ (2) = 43.29, *p* < 0.001									
Education level	6991		3249		2211		1038							
Any qualification ^d^	4829	69.1	2276	70.1	1508	68.2	768	74.0	Ref		Ref		Ref	
No qualifications	2162	30.9	973	29.9	703	31.8	270	26.0	0.93	0.84–1.03	0.95	0.84–1.06	0.89	0.76–1.05
	χ^2^ (1) = 1.00, n.s.				χ^2^ (2) = 12.12, *p* < 0.01									
Townsend index ^e^	6968		3245		2211		1034							
Score < 0	5859	84.1	2786	85.9	1910	86.4	876	84.7	Ref		Ref		Ref	
Score > 0	1109	15.9	459	14.1	301	13.6	158	15.3	0.93	0.82–1.05	0.88	0.76–1.02	1.04	0.86–1.26
	χ^2^ (1) = 5.34, *p* < 0.05				χ^2^ (2) = 6.85, *p* < 0.05									
Smoking	6951		3226		2197		1029							
Never	2295	33.0	1111	34.4	730	33.2	381	37.0	Ref		Ref		Ref	
Former	3756	54.0	1872	58.0	1309	59.6	563	54.7	0.95	0.87–1.05	0.97	0.87–1.08	0.91	0.79–1.05
Current	900	12.9	243	7.5	158	7.2	85	8.3	**0.59**	0.50–0.69	**0.58**	0.48–0.70	**0.61**	0.47–0.79
	χ^2^ (2) = 65.22, *p* < 0.001				χ^2^ (4) = 71.95, *p* < 0.001									
Physical activity ^f^	6991		3249		2211		1038							
(Moderately) active	3060	43.8	1475	45.4	1000	45.2	475	45.8	Ref		Ref		Ref	
(Moderately) inactive	3931	56.2	1774	54.6	1211	54.8	563	54.2	**0.82**	0.75–0.90	**0.82**	0.74–0.91	**0.81**	0.71–0.93
	χ^2^ (1) = 2.38, n.s.				χ^2^ (2) = 2.46, n.s.									
Start 7dDD ^g^	6994		3252		2215		1037							
Spring/Summer	3507	50.1	1541	47.4	1066	48.1	475	45.8	Ref		Ref		Ref	
Autumn/Winter	3487	49.9	1711	52.6	1149	51.9	562	54.2	**1.12**	1.03–1.22	1.09	0.98–1.20	**1.20**	1.05–1.37
	χ^2^ (1) = 6.75, *p* < 0.01				χ^2^ (2) = 8,27, *p* < 0.05									

NSU, Non-supplement User; SU, Supplement User; CLO, cod liver oil; χ^2^, Chi-squared test; OR, odds ratio; CI, confidence interval; Ref, Reference category. ^a^ Not married included the categories (NSU/SU): single (*n* = 290/128), widowed (*n* = 213/106), separated (*n* = 69/19) and divorced (*n* = 286/135); ^b^ Non-manual included the categories (NSU/SU): professional (*n* = 544/222), managerial (*n* = 2531/1315), skilled non-manual (*n* = 860/424); ^c^ Manual included the categories (NSU/SU): skilled manual (*n* = 1787/760), semi-skilled (*n* = 934/397) and non-skilled (*n* = 225/79); ^d^ Any qualification included (NSU/SU): O-level (*n* = 587/291), A-level (*n* = 3177/1518), Degree or equivalent (*n* = 1065/467); ^e^ Townsend index score < 0 means district in which the participant lives is more affluent than the mean in England; score > 0 means a district in which the participant lives is more deprived than the mean in England; ^f^ Included the categories (NSU/SU): active (1467/705), moderately active (1593/770), moderately inactive (1685/837), inactive (2246/937); ^g^ Created from first date in the diary; Spring: March-May, Summer: June-August, Autumn: September-November, Winter: December-February.

**Table 2:** Characteristics of EPIC-Norfolk participants (women only) according to supplement status (NSU/SU) and supplement subgroup (NSU/SU+CLO/SU-CLO). Analysis are shown unadjusted (Chi-squared test). Logistic regression (NSU/SU) and multinomial logistic regression (NSU/SU+CLO/SU-CLO) were adjusted for all variables in this table *(n* = 12,262). Boldly printed OR were statistically significant findings.

*WOMEN*	*NSU N*	*%*	*SU N*	*%*	*SU+CLO N*	*%*	*SU-CLO N*	*%*	*SU vs. NSU OR*	*95% CI*	*SU+CLO vs. NSU OR*	*95% CI*	*SU-CLO vs. NSU OR*	*95% CI*
Age	7056		5736		3389		2347							
<=50 years	1833	26.0	1356	23.6	611	18.0	745	31.7	1.02	1.00–1.04	**1.09**	1.06–1.12	**0.93**	0.90–0.95
>50–60 years	2185	31.0	1862	32.5	1107	32.7	755	32.2	OR represents the % change in odds of being					
>60–70 years	2100	29.8	1792	31.2	1170	34.5	622	26.5	a SU for every 5 year increment in age					
>70 years	938	13.3	726	12.7	501	14.8	225	9.6						
	χ^2^ (3) = 12.43 *p* < 0.01				χ^2^ (6) = 175.26 *p* < 0.001									
Marital status	7025		5691		3363		2328							
Married	5400	76.9	4315	75.8	2519	74.9	1796	77.1	Ref		Ref		Ref	
Not married ^a^	1625	23.1	1376	24.2	844	25.1	532	22.9	1.06	0.98–1.16	1.06	0.96–1.17	1.06	0.94–1.20
	χ^2^ (1) = 1.91 n.s.				χ^2^ (2) = 5.75 n.s.									
Social class	6865		5612		3299		2313							
Non-Manual ^b^	4063	59.2	3610	64.3	2049	62.1	1561	67.5	Ref		Ref		Ref	
Manual ^c^	2802	40.8	2002	35.7	1250	37.9	752	32.5	**0.84**	0.78–0.91	**0.90**	0.82–0.99	**0.76**	0.69–0.85
	χ^2^ (1) = 34.48 *p* < 0.001				χ^2^ (2) = 51.09 *p* < 0.001									
Education level	7052		5732		3386		2346							
Any qualification ^d^	3942	55.9	3423	59.7	1869	55.2	1554	66.2	Ref		Ref		Ref	
No qualifications	3110	44.1	2309	40.3	1517	44.8	792	33.8	**0.91**	0.84–0.99	1.02	0.93–1.11	**0.77**	0.69–0.86
	χ^2^ (1) = 18.88 *p* < 0.001				χ^2^ (2) = 88.08 *p* < 0.001									
Townsend index ^e^	7030		5710		3375		2335							
Score <0	5845	83.1	4845	84.9	2851	84.5	1994	85.4	Ref		Ref		Ref	
Score >0	1185	16.9	865	15.1	524	15.5	341	14.6	0.91	0.82–1.01	0.91	0.81–1.02	0.91	0.80–1.05
	χ^2^ (1) = 6.80 *p* < 0.01				χ^2^ (2) = 7.67 *p* < 0.05									
Smoking	6992		5680		3350		2330							
														
Never	3.949	56.5	3260	57.4	1939	57.9	1321	56.7	Ref		Ref		Ref	
Former	2179	31.2	1930	34.0	1162	34.7	768	33.0	1.08	1.00–1.17	1.07	0.98–1.17	1.09	0.98–1.21
Current	864	12.4	490	8.6	249	7.4	24	10.3	**0.71**	0.63–0.80	**0.61**	0.53–0.72	**0.85**	0.72–0.99
	χ^2^ (2) = 48.93 *p* < 0.001				χ^2^ (4) = 61.43 *p* < 0.001									
Physical activity ^f^	7052		5732		3386		2346							
(Moderately) active	2561	36.3	2259	39.4	1282	37.9	977	41.6	Ref		Ref		Ref	
(Moderately) inactive	4491	63.7	3473	60.6	2104	62.1	1369	58.4	**0.87**	0.80–0.94	**0.87**	0.80–0.96	**0.86**	0.77–0.95
	χ^2^ (1) = 12.89 *p* < 0.001				χ^2^ (2) = 21.34 *p* < 0.001									
Start 7dDD ^g^	7056		5736		3389		2347							
Spring/Summer	3662	51.9	2765	48.2	1640	48.4	1125	47.9	Ref		Ref		Ref	
Autumn/Winter	3394	48.1	2971	51.8	1749	51.6	1222	52.1	**1.16**	1.08–1.25	**1.15**	1.06–1.26	**1.17**	1.07–1.29
	χ^2^ (1) = 17.28 *p* < 0.001				χ^2^ (2) = 17.39 *p* < 0.001									

NSU, Non-supplement User; SU, Supplement User; CLO, cod liver oil; χ ^2^, Chi-squared test; CI, confidence interval; Ref, Reference category. ^a^ Not married includes the categories (NSU/SU): single (*n* = 281/230), widowed (*n* = 806/670), separated (*n* = 67/65) and divorced (*n* = 471/411); ^b^ Non-manual includes the categories (NSU/SU): professional (*n* = 421/372), managerial (*n* = 2343/2026), skilled non-manual (*n* = 1299/1212); ^c^ Manual includes the categories (NSU/SU): skilled manual (*n* = 1511/1115), semi-skilled (*n* = 971/702) and non-skilled (*n* = 320/185); ^d^ Any qualification included (NSU/SU): O-level (*n* = 830/640), A-level (*n* = 2363/2163), Degree or equivalent (*n* = 749/620); ^e^ Townsend index score < 0 means district in which the participant lives is more affluent than the mean in England; score > 0 means a district in which the participant lives is more deprived than the mean in England; ^f^ Included the categories (NSU/SU): active (1012/954), moderately active (1549/1305), moderately inactive (2186/1924), inactive (2305/1549); ^g^ Created from first date in the diary; Spring: March-May, Summer: June-August, Autumn: September-November, Winter: December-February.

### 3.3. Food Choices of Supplement Users

SU in general consumed significantly more fruit, vegetables and fatty fish and less red and processed meat than NSU (Table 3). In both men and women, the lower intake of red and processed meats amongst SU in general appeared to be mainly driven by the SU-CLO group, which had a significantly lower intake compared to the SU+CLO group (*p* < 0.025). Although there were no associations between alcohol consumption and supplement use in men, we observed a higher proportion of alcohol consumers among women using supplements, particularly the SU-CLO group, as well as an increment in their median weekly intake (*p* < 0.025).

### 3.4. Health and Supplement Use

BMI was associated with both age and supplement use. The mean (95% CI), age-adjusted BMI for male NSU was 26.6 (26.5–26.7) kg/m^2^, for SU+CLO 26.3 (26.2–26.4) kg/m^2^ and for SU-CLO 26.1 (25.9–26.2) kg/m^2^ (F = 15.6 [2;10217], *p* < 0.001). Among women, the association between supplement use and BMI was stronger; the mean BMI for female NSU was 26.4 (26.3–26.5) kg/m^2^, for SU+CLO 25.9 (25.7–26.0) kg/m^2^ and for SU-CLO 25.6 (25.4–25.8) kg/m^2^ (F = 42.4 [2;12750], *p* < 0.001).

In this *cross-sectional* study, the use of different types of dietary supplements was associated with self-reported illnesses (Table 4). For participants who reported having had benign growths, the odds of being a SU-CLO increased by 36% in men and 35% in women compared to NSU. Diseases affecting the heart and circulation were *negatively* associated with CLO supplement use and not associated with non-CLO supplement use. Men who reported having had a heart attack had a 42% lower odds of using CLO compared to men free of a prevalent heart attack; women who reported having been diagnosed with diabetes had a 50% reduced odds of being a SU+CLO. Participants who reported diseases that affect bone health were reporting more supplement use. Women who reported arthritis had a 60% increased odds of using CLO and 15% increased use of other types of supplements; similar results for CLO use were found for men. Women who reported osteoporosis had a 58% increased odds of using a non-CLO supplement.

**Table 3:** Comparison of food group intake distributions between Non-Supplement Users and Supplement Users (NSU/SU) and between SU subgroups (NSU/SU+CLO/SU-CLO) in the EPIC-Norfolk study.

*Food groups*	*NSU Median*	*IQR*	*SU Median*	*IQR*	*SU+CLO Median*	*IQR*	*SU-CLO Median*	*IQR*	*p-value ^a^* *NSU vs. SU*	*p-value ^b^* *NSU vs. SU+CLO vs. SU-CLO*
MEN (n)	6994		3252		2215		1037			
Fruit (g/day)	127	60–212	161	86–253	161	89–257	158	79–244	*p* < 0.001	*p* < 0.001
Vegetables (g/day)	139	99–188	145	106–198	146	108–197	145	102–202	*p* < 0.001	*p* < 0.001
Meat										
Red (g/day)	38	20–59	33	16–54	*34	16–55	31	14–52	*p* < 0.001	*p* < 0.001
White (g/day)	22	7–40	22	7–40	22	8–40	21	6–40	n.s.	n.s.
Processed (g/day)	25	13–40	22	11–37	*23	12–38	21	9–35	*p* < 0.001	*p* < 0.001
Fish										
White(g/day)	16	0–25	16	0–27	16	0–27	15	0–27	*p* < 0.01	*p* < 0.01
Fatty (g/day)	1	0–19	8	0–24	7	0–24	8	0–24	*p* < 0.001	*p* < 0.001
Alcoholic beverages (units/diary)	8.1	1.2–21.0	8.2	1.2–21.1	8.1	1.3–20.9	8.4	0.9–21.8	n.s.	n.s.
Alcohol consumers only (units/diary) ^c^	13.0	5.3–25.5	13.0	5.4–25.5	12.8	5.1–25.1	13.9	6.0–26.8	n.s.	n.s.
WOMEN (n)	7056		5736		3389		2347			
Fruit (g/day)	151	83–238	180	107–267	*183	110–269	174	104–263	*p* < 0.001	*p* < 0.001
Vegetables (g/day)	136	97–183	145	107–194	145	108–192	146	105–197	*p* < 0.001	*p* < 0.001
Meat										
Red (g/day)	27	12–45	24	9–41	*25	10–42	23	7–40	*p* < 0.001	*p* < 0.001
White (g/day)	18	5–34	19	6–35	19	5–36	19	6–35	*p* < 0.05	n.s.
Processed (g/day)	16	7–27	14	6–25	*15	6–25	14	4–24	*p* < 0.001	*p* < 0.001
Fish										
White (g/day)	12	0–21	12	0–22	*13	0–23	11	0–21	n.s.	*p* < 0.001
Fatty (g/day)	3	0–16	6	0–20	6	0–21	6	0–19	*p* < 0.001	*p* < 0.001
Alcoholic beverages (units/diary)	2.1	0.0–9.2	3.3	0–10.3	*3.1	0.0–9.8	3.9	0.0–11.2	*p* < 0.001	*p* < 0.001
Alcohol consumers only (units/diary) ^d^	6.9	3.0–14.6	7.3	3.3–14.7	*7.0	3.2–14.1	7.7	3.5–15.2	n.s.	*p* < 0.05

NSU, Non-supplement User; SU, Supplement User; CLO, cod liver oil; IQR, Inter Quartile Range. ^a^ Differences between groups tested using Mann-Whitney U test; ^b^ Differences between groups tested using Kruskal-Wallis test and if significant, followed by Mann-Whitney U test to test for differences in SU subgroups. *p*-values < 0.025 were considered significant (Bonferroni correction applied: 0.05/2, marked with an * in the SU+CLO column); ^c^ Men: NSU *n* = 5411 (77%), SU+CLO *n* = 1748 (79%), SU-CLO *n* = 799 (77%); ^d^ Women: NSU *n* = 4352 (62%) , SU+CLO *n* = 2233 (66%), SU-CLO *n* = 1611 (69%); * *p*-value < 0.025 (see also footnote b).

**Table 4:** Differences in self-reported health between non-supplement users (NSU) and supplement user subgroups (SU+CLO, SU-CLO) in EPIC-Norfolk.

*Health condition*	*Answer category*	*N*	*%*	*NSU N*	*%*	*SU+CLO N*	*%*	*SU-CLO N*	*%*	*SU+CLO vs NSU* *OR ^a^*	*95% C.I.*	*SU-CLO vs. NSU* *OR ^a^*	*95% C.I.*
MEN		10,247		6994	68.3	2215	21.6	1038	10.1				
Mean age (SD)				58.9	9.3	61.8	8.6	59.4	9.3	**1.19**	1.16–1.22	1.03	0.99–1.07
Benign growth	Yes	993	9.7	634	9.1	234	10.6	125	12.1	1.12	0.96–1.32	**1.36**	1.11–1.67
	No	9235	90.3	6348	90.9	1975	89.4	912	87.9	Ref		Ref	
Cancer	Yes	405	4.0	259	3.7	94	4.3	52	5.0	0.98	0.76–1.24	1.33	0.98–1.89
	No	9830	96.0	6727	96.3	2117	95.7	986	95.0	Ref		Ref	
Heart attack	Yes	560	5.5	409	5.9	94	4.3	57	5.5	**0.58**	0.46–0.74	0.90	0.68–1.20
	No	9668	94.5	6573	94.1	2116	95.7	979	94.5	Ref		Ref	
Stroke	Yes	189	1.8	139	2.0	36	1.6	14	1.4	**0.65**	0.45–0.94	0.64	0.37–1.12
	No	10,041	98.2	6846	98.0	2173	98.4	1022	98.6	Ref		Ref	
High blood pressure	Yes	1469	14.4	979	14.0	328	14.9	162	15.6	0.92	0.80–1.05	1.11	0.92–1.33
	No	8753	85.6	5999	86.0	1879	85.1	875	84.4	Ref		Ref	
Diabetes	Yes	333	3.3	236	3.4	69	3.1	28	2.7	0.78	0.59–1.03	0.77	0.52–1.14
	No	9898	96.7	6747	96.6	2141	96.9	1010	97.3	Ref		Ref	
Arthritis	Yes	1932	18.9	1137	16.3	604	27.4	191	18.5	**1.72**	1.53–1.93	1.14	0.96–1.35
	No	8282	81.1	5837	83.7	1602	72.6	843	81.5	Ref		Ref	
Osteoporosis	Yes	60	0.6	42	0.6	12	0.5	6	0.6	0.75	0.39–1.43	0.93	0.39–2.19
	No	10,166	99.4	6940	99.4	2194	99.5	1032	99.4	Ref		Ref	
													
WOMEN		12792		7056	55.2	3389	26.5	18.3	2347				
Mean age (SD)				58.6	9.4	60.0	8.8	56.9	9.2	**1.09**	1.07–1.12	**0.91**	0.88–0.93
Benign growth	Yes	2448	19.2	1227	17.4	703	20.8	518	22.1	**1.25**	1.12–1.38	**1.35**	1.21–1.52
	No	10,304	80.8	5812	82.6	2671	79.2	1821	77.9	Ref		Ref	
Cancer	Yes	883	6.9	468	6.6	258	7.6	157	6.7	1.11	0.94–1.30	1.07	0.89–1.30
	No	11,898	93.1	6582	93.4	3127	92.4	2189	93.3	Ref		Ref	
Heart attack	Yes	168	1.3	105	1.5	39	1.2	24	1.0	**0.66**	0.46–0.96	0.82	0.52–1.28
	No	12,608	98.7	6941	98.5	3345	98.8	2322	99.0	Ref		Ref	
Stroke	Yes	127	1.0	86	1.2	26	0.8	15	0.6	**0.55**	0.35–0.86	0.61	0.35–1.06
	No	12,651	99.0	6962	98.8	3358	99.2	2331	99.4	Ref		Ref	
High blood pressure	Yes	1840	14.4	1067	15.1	483	14.3	290	12.4	**0.84**	0.74–0.94	0.89	0.77–1.03
	No	10,926	85.6	5977	84.9	2899	85.7	2050	87.6	Ref		Ref	
Diabetes	Yes	205	1.6	139	2.0	37	1.1	29	1.2	**0.50**	0.35–0.73	0.69	0.46–1.03
	No	12,571	98.4	6908	98.0	3347	98.9	2316	98.8	Ref		Ref	
Arthritis	Yes	3495	27.4	1717	24.4	1195	35.4	583	25.0	**1.60**	1.46–1.76	**1.15**	1.03–1.28
	No	9247	72.6	5316	75.6	2180	65.6	1751	75.0	Ref		Ref	
Osteoporosis	Yes	340	2.7	164	2.3	100	3.0	76	3.2	1.17	0.91–1.51	**1.58**	1.20–2.09
	No	12,418	97.3	6877	97.7	3276	97.0	2265	96.8	Ref		Ref	

NSU, Non-supplement User; SU, Supplement User; CLO, cod liver oil; OR, odds ratio, CI, confidence interval. ^a^ Age-adjusted OR (per 5 year) using multinomial logistic regression. Boldly printed OR were statistically significant findings.

## 4. Discussion

Supplement use in EPIC-Norfolk is more prevalent among women and is associated with not smoking, a higher social class, higher physical activity levels and more favourable eating habits. These SU characteristics were found to be stronger for subgroups of SU, than for SU in general. Moreover, a participant’s self-report of medical conditions at baseline was associated with subgroups of supplements, with CLO supplements being strongly positively associated with arthritis and negatively associated with cardiovascular conditions.

The associations found between supplement use in general and socio-demographic variables are in line with previous findings from a UK survey and cohort studies [4,5,21]. Our finding of more and stronger associations in women compared to men, has also been observed in the MRC National Survey of Health and Development [4]. However, important socio-demographic differences exist within SU. For example, in our study social class appeared to be mainly associated with SU-CLO use in men and women’s education was only associated with SU-CLO use and not SU+CLO use. Also, while most “unhealthy behaviours” were less prevalent among SU, exceptions were smoking and alcohol consumption among women in the SU-CLO group. The Norwegian Women and Cancer (NoWAC) study grouped participants into categories by frequency of consumption of CLO use [33]. Their average age of 45 years was 15 years lower than in EPIC-Norfolk; even so, participants’ age was positively associated with daily CLO consumption, as well as being an ex-smoker and being more physically active. Again, this stresses the different possible confounders within subgroups of SU.

In EPIC-Norfolk, SU, particularly the SU+CLO group, were found to have a higher consumption of fruit, vegetables and fatty fish and especially the SU-CLO group had a lower consumption of red and processed meat compared to NSU. These associations are comparable with other studies [4,5,21,33] and are indicative that SU are a group of people who are least likely to need supplements. Although SU have in general been characterised as “healthy eating” consumers, this might not necessarily be so [15,34]. A longitudinal study in Switzerland found that 21% of daily/weekly vitamin and mineral SU were clustered around a “healthy” food pattern (16% among NSU); whereas 31% of daily/weekly vitamin and mineral SU consumed an “unhealthy” food pattern (compared to 39% in NSU); the SU were also found to have the most positive attitude towards fortification and could have used supplements as a means of compensation. In the current analysis, only a limited set of foods were compared between NSU and SU in order to avoid multiple testing, but future analyses could compare clusters of a greater variety of foods.

In this cross-sectional analysis, participants who reported having had benign growths were more likely to report non-CLO supplement use. Cancer was not associated with supplement use in the EPIC-Norfolk study, contrary to what has been found in the UK Women’s Cohort Study [35]. Also the VITAL cohort [18] reports significant associations between high dose vitamin E and cancer in women as well as a study among cancer survivors [36], where only vitamin use, but not mineral, herbal or other types of supplement use, was associated with cancer. In the VITAL cohort, the number of supplements consumed among women with a medical condition was higher than in men; however, the associations between supplement use and medical conditions were stronger in *men* [18]. It was suggested that women might use supplements to prevent illness, whereas men might start to take supplements after diagnosis. In EPIC-Norfolk, the associations between supplement use and medical conditions were of similar strength for men and women, but data collected at later health examinations will be able to answer important questions related to the onset of illness and the starting or stopping of supplement use. Although the time between diagnosis and the start of the use of dietary supplements is also of importance since participants might make a change in their habits shortly after diagnosis, but return to their former habits after some time has passed [36], the surveys in EPIC-Norfolk might not be frequent enough to capture these changes.

A limitation of our analysis is the stratification of results into SU+CLO and SU-CLO groups, since this is likely to have underestimated the heterogeneity among the SU-CLO subgroup. A recent analysis of the Hertfordshire Cohort Study (HCS) used cluster analysis to describe five groups of SU; however, plant and fish oils were grouped together [15]. The aim of our analysis was to study possible confounding variables of participants consuming fish and CLO supplements. SU+CLO reported more illnesses such as arthritis and less (symptoms of) cardiovascular disease and stroke, contrary to what is reported in the HCS [15]. A UK survey [5] and a survey among 65-98 year old Australians [24,25] however found similar associations to EPIC-Norfolk. The data collected at later health examinations, will have to be taken into account before causal inferences between CLO and cardiovascular diseases can be made, especially since meta-analyses have not shown benefits [11,12]. The fact that CLO is positively associated with age, and that it is more likely to be taken on a daily basis, makes the SU+CLO subgroup of particular interest to investigate further since exposure to CLO is likely to have been for an extended period of time and follow-up time in this prospective cohort is by now two decades, contrary to trials. The nutrients of these supplements are quantified in the ViMiS database where missing values for omega-3 fatty acids were completed, and units of measure were made compatible for food and supplement sources enabling the calculation of a “total nutrient exposure” in a detailed way [8,37]. The wide range of endpoints collected will enable us to look at potential positive as well as harmful effects of CLO.

## 5. Conclusions

Significant socio-demographic associations were found in this study with weaker and fewer associations in SU+CLO than in SU-CLO group, especially among men. Associations between supplement use and age, smoking, social class and education were strong, but not uniform across all SU or between sexes. Participants, who had prevalent heart attack or stroke, were less likely to report CLO supplements; however, self-reported arthritis was associated with increased CLO use. The differences we found between subgroups of SU provide important information that will be necessary for later endpoint analysis of this and other studies, since confounding by indication as well as lifestyle confounders will need to be taken into account depending on the type of supplement consumed.

## References

[1] Rajakumar K. (2003). Vitamin D, cod-liver oil, sunlight, and rickets: A historical perspective. Pediatrics.

[2] Bates C.J., Prentice A., van der Pols J.C., Walmsley C., Pentieva K.D., Finch S., Smithers G., Clarke P.C. (1998). Estimation of the use of dietary supplements in the National Diet and Nutrition Survey: People aged 65 years and Over. An observed paradox and a recommendation. Eur. J. Clin. Nutr..

[3] Henderson L., Gregory J., Swan G. (2002). The National Diet & Nutrition Survey: Adults Aged 19 to 64 Years. Types and Quantities of Foods Consumed.

[4] McNaughton S.A., Mishra G.D., Paul A.A., Prynne C.J., Wadsworth M.E.J. (2005). Supplement use is associated with health status and health-related behaviors in the 1946 British birth cohort. J. Nutr..

[5] Harrison R.A., Holt D., Pattison D.J., Elton P.J. (2004). Are those in need taking dietary supplements? A survey of 21 923 adults. Br. J. Nutr..

[6] Bates B., Lennox A., Bates C., Swan G. (2011). National Diet and Nutrition Survey. Headline Results from Years 1 and 2 (Combined) of the Rolling Programme (2008/2009–2009/10).

[7] Lentjes M.A.H., Welch A.A., Luben R.N., Khaw K.-T. (2013). Differences in dietary supplement use and secular and seasonal trends assessed using three different instruments in the EPIC-Norfolk population study. J. Diet. Suppl..

[8] Lentjes M.A.H., Bhaniani A., Mulligan A.A., Khaw K.-T., Welch A.A. (2011). Developing a database of vitamin and mineral supplements (ViMiS) for the Norfolk arm of the European Prospective Investigation into Cancer (EPIC-Norfolk). Public Health Nutr..

[9] Brasky T.M., Lampe J.W., Potter J.D., Patterson R.E., White E. (2010). Specialty supplements and breast cancer risk in the VITamins And Lifestyle (VITAL) Cohort. Cancer Epidemiol. Biomark. Prev..

[10] Skeie G., Braaten T., Hjartåker A., Brustad M., Lund E. (2009). Cod liver oil, other dietary supplements and survival among cancer patients with solid tumours. Int. J. Cancer.

[11] Chowdhury R., Stevens S., Gorman D., Pan A., Warnakula S., Chowdhury S., Ward H., Johnson L., Crowe F., Hu F.B. (2012). Association between fish consumption, long chain omega 3 fatty acids, and risk of cerebrovascular disease: Systematic review and meta-analysis. BMJ.

[12] Rizos E.C., Ntzani E.E., Bika E., Kostapanos M.S., Elisaf M.S. (2012). Association between omega-3 fatty acid supplementation and risk of major cardiovascular disease events: A systematic review and meta-analysis. JAMA.

[13] Scientific Advisory Committee on Nutrition (SACN) (2007). Update on Vitamin D.

[14] Mulholland C.A., Benford D.J. (2007). What is known about the safety of multivitamin-multimineral supplements for the generally healthy population? Theoretical basis for harm. Am. J. Clin. Nutr..

[15] Denison H.J., Jameson K.A., Syddall H.E., Dennison E.M., Cooper C., Sayer A.A., Robinson S.M. (2012). Patterns of dietary supplement use among older men and women in the UK: Findings from the Hertfordshire Cohort Study. J. Nutr. Health Aging.

[16] White E., Patterson R.E., Kristal A.R., Thornquist M., King I., Shattuck A.L., Evans I., Satia-Abouta J., Littman A.J., Potter J.D. (2004). VITamins And Lifestyle cohort study: Study design and characteristics of supplement users. Am. J. Epidemiol..

[17] Hoggatt K.J. (2003). Commentary: Vitamin supplement use and confounding by lifestyle. Int. J. Epidemiol..

[18] Satia-Abouta J., Kristal A.R., Patterson R.E., Littman A.J., Stratton K.L., White E. (2003). Dietary supplement use and medical conditions: The VITAL study. Am. J. Prev. Med..

[19] Gunther S., Patterson R.E., Kristal A.R., Stratton K.L., White E. (2004). Demographic and health-related correlates of herbal and specialty supplement use. J. Am. Diet. Assoc..

[20] Expert group on vitamins and minerals (2003). Safe Upper Levels for Vitamins and Minerals.

[21] Kirk S.F., Cade J.E., Barrett J.H., Conner M. (1999). Diet and lifestyle characteristics associated with dietary supplement use in women. Public Health Nutr..

[22] Skeie G., Braaten T., Hjartåker A., Lentjes M., Amiano P., Jakszyn P., Pala V., Palanca A., Niekerk E.M., Verhagen H. (2009). Use of dietary supplements in the European Prospective Investigation into Cancer and Nutrition calibration study. Eur. J. Clin. Nutr..

[23] Patterson R.E., White E., Kristal A.R., Neuhouser M.L., Potter J.D. (1997). Vitamin supplements and cancer risk: The epidemiologic evidence. Cancer Causes Control.

[24] Brownie S., Rolfe M. (2004). Health characteristics of older Australian dietary supplement users compared to non-supplement users. Asia Pac. J. Clin. Nutr..

[25] Brownie S. (2006). Predictors of dietary and health supplement use in older Australians. Aust. J. Adv. Nurs..

[26] Day N., Oakes S., Luben R., Khaw K.T., Bingham S., Welch A., Wareham N. (1999). EPIC-Norfolk: Study design and characteristics of the cohort. European Prospective Investigation of Cancer. Br. J. Cancer.

[27] Wareham N.J., Jakes R.W., Rennie K.L., Schuit J., Mitchell J., Hennings S., Day N.E. (2003). Validity and repeatability of a simple index derived from the short physical activity questionnaire used in the European Prospective Investigation into Cancer and Nutrition (EPIC) study. Public Health Nutr..

[28] Shohaimi S., Luben R., Wareham N., Day N., Bingham S., Welch A., Oakes S., Khaw K.-T. (2003). Residential area deprivation predicts smoking habit independently of individual educational level and occupational social class. A cross sectional study in the Norfolk cohort of the European Investigation into Cancer (EPIC-Norfolk). J. Epidemiol. Commun. Health.

[29] Bingham S.A., Welch A.A., McTaggart A., Mulligan A.A., Runswick S.A., Luben R., Oakes S., Khaw K.T., Wareham N., Day N.E. (2001). Nutritional methods in the European Prospective Investigation of Cancer in Norfolk. Public Health Nutr..

[30] Welch A.A., McTaggart A., Mulligan A.A., Luben R., Walker N., Khaw K.T., Day N.E., Bingham S.A. (2001). DINER (Data Into Nutrients for Epidemiological Research)—A new data-entry program for nutritional analysis in the EPIC-Norfolk cohort and the 7-day diary method. Public Health Nutr..

[31] Lentjes M.A.H., McTaggart A., Mulligan A.A., Powell N.A., Parry-Smith D., Luben R.N., Bhaniani A., Welch A.A., Khaw K.-T. (2014). Dietary intake measurement using 7 d diet diaries in British men and women in the European Prospective Investigation into Cancer-Norfolk study: A focus on methodological issues. Br. J. Nutr..

[32] World Cancer Research Fund/American Institute for Cancer Research (2007). Food, Nutrition, Physical Activity, and the Prevention of Cancer: A Global Perspective.

[33] Brustad M., Braaten T., Lund E. (2004). Predictors for cod-liver oil supplement use--the Norwegian Women and Cancer Study. Eur. J. Clin. Nutr..

[34] Van der Horst K., Siegrist M. (2011). Vitamin and mineral supplement users. Do they have healthy or unhealthy dietary behaviours?. Appetite.

[35] Hutchinson J., Burley V.J., Greenwood D.C., Thomas J.D., Cade J.E. (2011). High-dose vitamin C supplement use is associated with self-reported histories of breast cancer and other illnesses in the UK Women’s Cohort Study. Public Health Nutr..

[36] Miller M.F., Bellizzi K.M., Sufian M., Ambs A.H., Goldstein M.S., Ballard-Barbash R. (2008). Dietary supplement use in individuals living with cancer and other chronic conditions: A population-based study. J. Am. Diet. Assoc..

[37] Lentjes M.A.H., Mulligan A.A., Welch A.A., Bhaniani A., Luben R.N., Khaw K.-T. (2014). Contribution of cod liver oil-related nutrients (vitamins A, D, E and eicosapentaenoic acid and docosahexaenoic acid) to daily nutrient intake and their associations with plasma concentrations in the EPIC-Norfolk cohort. J. Hum. Nutr. Diet..

